# Disulfide loop cleavage of *Legionella pneumophila* PlaA boosts lysophospholipase A activity

**DOI:** 10.1038/s41598-017-12796-4

**Published:** 2017-11-24

**Authors:** Christina Lang, Miriam Hiller, Antje Flieger

**Affiliations:** Division of Enteropathogenic Bacteria and Legionella (FG11), Robert Koch-Institut, Burgstr. 37, D-38855 Wernigerode, Germany

## Abstract

*L*. *pneumophila*, an important facultative intracellular bacterium, infects the human lung and environmental protozoa. At least fifteen phospholipases A (PLA) are encoded in its genome. Three of which, namely PlaA, PlaC, and PlaD, belong to the GDSL lipase family abundant in bacteria and higher plants. PlaA is a lysophospholipase A (LPLA) that destabilizes the phagosomal membrane in absence of a protective factor. PlaC shows PLA and glycerophospholipid: cholesterol acyltransferase (GCAT) activities which are activated by zinc metalloproteinase ProA via cleavage of a disulphide loop. In this work, we compared GDSL enzyme activities, their secretion, and activation of PlaA. We found that PlaA majorly contributed to LPLA, PlaC to PLA, and both substrate-dependently to GCAT activity. Western blotting revealed that PlaA and PlaC are type II-secreted and both processed by ProA. Interestingly, ProA steeply increased LPLA but diminished GCAT activity of PlaA. Deletion of 20 amino acids within a predicted disulfide loop of PlaA had the same effect. In summary, we propose a model by which ProA processes PlaA via disulfide loop cleavage leading to a steep increase in LPLA activity. Our results help to further characterize the *L*. *pneumophila* GDSL hydrolases, particularly PlaA, an enzyme acting in the *Legionella*-containing phagosome.

## Introduction

*Legionella pneumophila* is an important facultative intracellular bacterium, with protozoa as its natural host. It can also survive within biofilms in technical water systems. In humans, the bacterium causes severe damage of the lung, which may lead to pneumonia and fatalities. A steady increase of Legionnaires’ pneumonia has been noted in Europe during the last years resulting in about 7000 cases including more than 500 deaths in 2014^1^. Furthermore, outbreaks involving multitudes of cases have been frequently described^2^.

Phospholipases are important virulence factors of *L*. *pneumophila*, which can interfere with host cell processes and shape infection outcome. They are abundant in *L*. *pneumophila* with at least 15 different phospholipases A (PLAs), three phospholipases C, and one phospholipase D being encoded in the genome^3–10^. PLAs in *L*. *pneumophila* divide into three major groups, the patatin-like, the PlaB-like and the GDSL enzymes^3,11^. The latter are represented in many bacteria and also higher plants and include the following enzymatic spectrum: PLA, lysophospholipase A (LPLA), lipase, haemolytic, and glycerophospholipid: cholesterol acyltransferase (GCAT) activities^12,13^. One example is the *Aeromonas salmonicida* type II-secreted acyltransferase/PLA SatA, which is an extracellular lethal toxin for Atlantic salmon and active on fish erythrocytes^14–18^. Extracellular GCAT activity of SatA is triggered by the bacterial serine protease AspA. SatA can also be activated by trypsin, cleaving the toxin within a disulphide loop flanked by residues cysteine 225 and cysteine 281^19,20^. Another example is the *Salmonella* pathogenicity island 2 (SPI2) type III-secreted effector SseJ, which also exhibits acyltransferase and PLA activities following activation by the host cell small GTPase RhoA^21–24^. SseJ together with SifA, another SPI2 effector, have been shown to regulate dynamics and integrity of the *Salmonella*-containing compartment. In the absence of SifA, membrane integrity of the *Salmonella*-containing vacuole is lost and the bacteria are released into the cytosol. An additional knock out of *sseJ* reverts this effect^24–27^.

**Figure 1:**
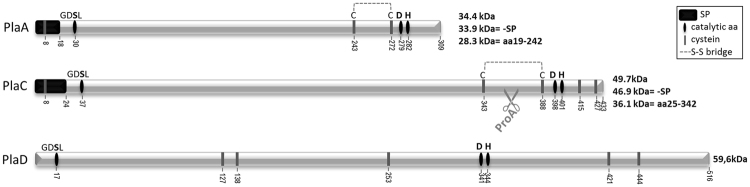
Schematic overview of *L*. *pneumophila* GDSL hydrolases PlaA, PlaC, and PlaD. Localization of putative catalytic amino acids of *L*. *pneumophila* PlaA, PlaC and PlaD as well as predicted signal peptides (SignalP 4.1 Server) and disulphide bonds (DiANNA 1.1 web server) of PlaA and PlaC are depicted. Abbreviations: SP—predicted signal peptide, -SP—variant after cleavage of signal peptide, aa—amino acid(s), S-S bridge—predicted disulphide bond, aa19-242 and aa25-342—PlaA- and PlaC-derived variants comprising amino acids 19 till 242 and 25 till 342, respectively, after loss of signal peptide and loss of C-terminal region after disulphide loop cleavage and reduction of disulphide bond.

*L*. *pneumophila* possesses three GDSL enzymes, PlaA, PlaC, and PlaD, which share a similar putative catalytic triade. However, they differ in protein size, protein organisation, and predicted signal peptides, indicating that they might have different properties and functions (Fig. 1)^3^. While the activity of PlaD has not yet been comprehensively defined, PlaA and PlaC indeed show distinct activities. PlaC predominantly exhibits GCAT and PLA activities^28,29^. Analysis of single GDSL enzyme knock out mutants showed that PlaC represents the only enzyme in *L*. *pneumophila* transferring palmitic acid from diacaylphospholipids to cholesterol and ergosterol. The *L*. *pneumophila* zinc metalloproteinase ProA highly promotes PlaC-derived GCAT and PLA activities. It directly processes a disulphide loop region in PlaC leading to enzyme activation. These data suggest that a disulphide loop inhibits PlaC GCAT activity until the protein is exported and ProA-activated. The three putative catalytic amino acids, serine 37, aspartate 398, and histidine 401, proved to be essential for all PlaC-associated activities^30^. PlaA is the major secreted LPLA^29,31,32^. PlaA, and to some extent PlaC, transfer the short chain fatty acid, propionic acid, to cholesterol and ergosterol^30^. However, whether an activation procedure supports PlaA activity has not been described.

Loss of specific activities in secretion mutants and the presence of a signal peptide indicate that PlaA and PlaC are secreted by the *L*. *pneumophila* type II secretion system Lsp. PlaC is further found in outer membrane vesicles (OMVs)^28,32–35^. Both OMVs and the type II-secreted protease ProA have been detected within the lumen of the *Legionella*-containing vacuole (LCV)^34,36^ and an influence of PlaA on the phagosomal membrane has been shown before. Specifically, PlaA promotes membrane destabilization of the LCV in the absence of the type IVB-secreted effector SdhA, resulting in the activation of host cell death pathway^37^. PlaA and PlaC have the potential to act on phagosomal lipids and further modify cholesterol-rich regions within mammalian cells, as it was previously shown for SseJ, a GCAT of *Salmonella enterica*. Therefore, *L*. *pneumophila* PlaA and PlaC may influence LCV receptor presentation, membrane organization and stability^21,38^.

Here, we aimed to characterize enzyme activity and a possible mode of activation for *L*. *pneumophila* PlaA. In a first step, we compared the contribution of the three *L*. *pneumophila* GDSL enzymes to bacterial PLA/LPLA and GCAT activities and their export into the bacterial culture supernatant. Then, we addressed the question whether ProA has an impact on enzyme activity of PlaA, as it was shown for PlaC. Indeed, ProA is an important factor in the processing of PlaA via disulphide loop cleavage thereby modulating its enzymatic activity.

## Results

### PlaA majorly contributes to LPLA activity, PlaC to PLA activity, and both substrate-dependently to GCAT activity of *L. pneumophila* culture supernatant

**Figure 2:**
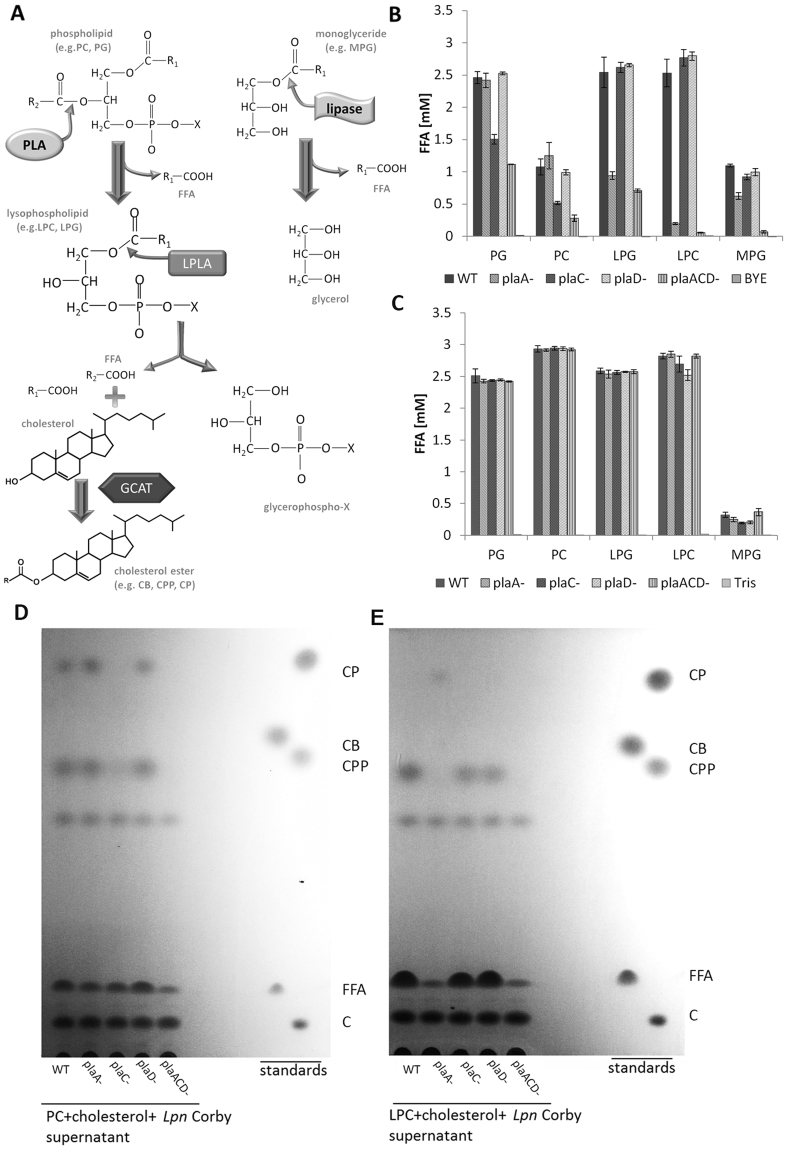
PlaA majorly contributes to LPLA, PlaC to PLA, and both substrate-dependently to GCAT activity of *L*. *pneumophila* culture supernatant. (**A**) Schematic overview of lipolytic activities of PlaA (PLA, LPLA, GCAT). Displayed are cleavage sites in phospholipids and monoglycerides as well as reaction products. R1 and R2 denote long chain fatty acid residues and X the polar headgroup. PLA and LPLA activities of culture supernatans (**B**) and cell lysates (**C**) from *L*. *pneumophila* Corby wild type and different knock out mutants (*plaA* ^−^, *plaC* ^−^, *plaD* ^−^ and *plaACD* ^−^) were determined via quantification of FFA after incubation for 16 h with the indicated lipids at 37 °C. Formation of different cholesterol esters by *L*. *pneumophila* culture supernatants was studied after incubation for 16 h at 37 °C with PC (**D**) and LPC (**E**) followed by lipid extraction and TLC. The results represent the means and standard deviations of duplicate reactions (**B**,**C**). All results shown are representative for at least two additional experiments. *CP*, cholesterol palmitate; *CB*, cholesterol butyrate; *CPP*, cholesterol propionate; *FFA*, free fatty acids; *C*, cholesterol.

To compare the contribution of the GDSL enzymes to different lipolytic activities in *L*. *pneumophila*, we analyzed single mutants and a triple mutant for fatty acid release and sterol ester formation after incubation with different lipid substrates (Fig. 2A). Figure 2B shows amounts of free fatty acids (FFA) liberated by culture supernatants where phosphatidylglycerol (PG) and phosphatidylcholine (PC) hydrolysis reveals PLA, lysophosphatidylglycerol (LPG) and lysophosphatidylcholine (LPC) LPLA and monoacylglycerol (MPG) lipase activities. As expected, wild type bacteria showed highest levels of activities towards all substrates. Reductions in secreted activities were noted for the *plaA*, the *plaC* and the *plaACD* triple mutant but not for the *plaD* mutant. Specifically, the *plaA* mutant was strongly reduced in LPG, LPC, and to some extent in MPG hydrolysis confirming major LPLA activity of PlaA. The *plaC* mutant showed reductions in PG and PC hydrolysis indicating contribution of PlaC to PLA activity. The *plaACD* mutant revealed lowest activities representing the additive lack of PlaA and PlaC. Nevertheless, hydrolysis of PG, PC and LPG was observed for the *plaACD* mutant supernatant revealing the presence of further secreted PLA/LPLA(s) in addition to the GDSL enzymes. PlaD under these conditions had no contribution to the secreted lipolytic activities (Fig. 2B). We next asked whether the three enzymes might have a role in cell-associated activities. However, no decreased activities of the mutants were found suggesting that cell-associated activities were independent of PlaA, PlaC or PlaD (Fig. 2C).

Further, we analyzed GCAT activity of *L*. *pneumophila* culture supernatants. Here we confirmed the importance of PlaC for transfer of fatty acids from PC to cholesterol, represented by the lack of cholesterolpalmitate (CP) and –propionate (CPP) generation by the *plaC* and *plaACD* mutants (Fig. 2D). When, however, LPC was used as a donor of palmitic acid, the wild type did not develop CP at all, indicating that under those conditions palmitic acid transfer is not favored by the GDSL enzymes. Interestingly, only the *plaA* mutant formed CP, implying that in the absence of PlaA, another enzyme, such as PlaC, may use LPC (which here was not reacted by PlaA) as a donor for palmitic acid (Fig. 2E). Wild type and the single mutants produced CPP, albeit with different efficiencies. Among the single mutants, the *plaA* mutant displayed the greatest reduction in the generation of CPP confirming a previous observation that PlaA contributes significantly to the CPP production in *L*. *pneumophila* ^30^. Neither CP nor CPP could be detected with the culture supernatant of the *plaACD* mutant, displaying exclusive contribution of the three GDSL enzymes to GCAT activity (Fig. 2D,E). To summarize, PlaA and PlaC activities in *L*. *pneumophila* culture supernatant showed clear differences. PlaA contributed majorly to LPLA activity and acyl transfer from lysophospholipids to cholesterol whereas PlaC revealed PLA activity and acyl transfer from diacylphospholipids to cholesterol.

### PlaA and PlaC are type II-secreted and zinc metalloproteinase ProA-processed

**Figure 3:**
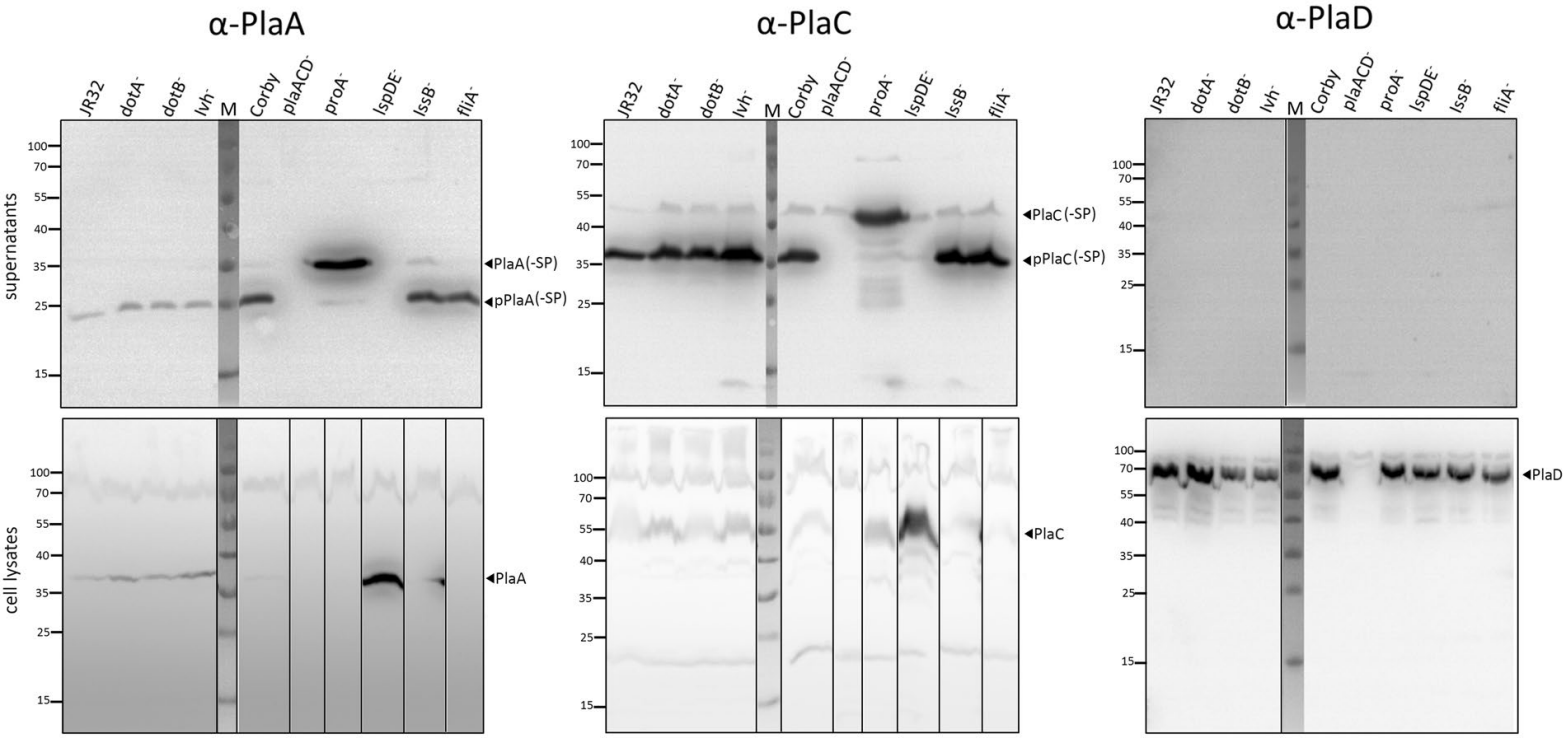
PlaA and PlaC are secreted via the type II secretion system Lsp and processed by the zinc metalloproteinase ProA. 10-fold concentrated supernatants (upper panel) and cell lysates (lower panel) of *L*. *pneumophila* Corby or JR32 wild types or isogenic mutant strains were applied to reducing SDS-PAGE, Western blot analysis and detection with α-PlaA, α-PlaC, and α-PlaD antibodies. Results are representative for at least two additional experiments. Molecular weight standards (M) are shown on the left side of each panel in kDa. Blot images were slightly cropped from all sides. α-PlaA and α-PlaC cell lysates blot images were vertically sliced to adjust the lanes to the corresponding supernatant blot image. Full length blots are presented in Supplementary Fig. S1. (-SP = without signal peptide; p = processed).

PlaA and PlaD were recombinantly expressed, purified, and polyclonal antibodies were raised as described earlier for PlaC^30^. Previously, secretion types of PlaA and PlaC have been deduced from the lack of enzymatic activities in the culture supernatants of defined secretion mutants^28,32^. The generation of specific antibodies provided us with a tool to directly detect the proteins in different bacterial fractions, and assess their secretion path and processing. To that end, we prepared culture supernatants and cell lysates from wild type, different secretion mutants and the *proA* mutant. After Western blot analysis, the pattern observed was quite similar for PlaA and PlaC. Both proteins were found in the supernatant of all strains but not in the *lspDE* mutant and the negative control (*plaACD* ^-^), confirming that PlaA and PlaC export is type II secretion system-dependent (Fig. 3). While PlaA in the supernatants was mostly present as a ~26–28 kDa protein, PlaC had a size of about 36 kDa. The latter represents the processed and ProA-activated form of PlaC^30^. Consequently, a larger protein version of about 47 kDa representing unprocessed PlaC could be detected in the supernatant of the *proA* mutant (Fig. 3, Fig. 1). PlaA was also present in a higher molecular form of ~34 kDa in the *proA* mutant supernatant suggesting its processing by ProA (Fig. 3, Fig. 1). Interestingly, PlaA and PlaC were not detected in substantial amounts in cell lysates of the different strains unless their secretion was prevented by an *lspDE* knock out (Fig. 3). On the contrary, PlaD was only detected in the cell lysates, except in the *plaACD* mutant, but not in culture supernatants (Fig. 3). In summary, we here provide evidence that PlaA and PlaC are Lsp type II-secreted and ProA-processed whereas PlaD remains cell associated.

### rPlaA shows LPLA, lipase, and GCAT activities which depend on the individual members of the catalytic triade: serine 30, aspartate 279, and histidine 282

**Figure 4:**
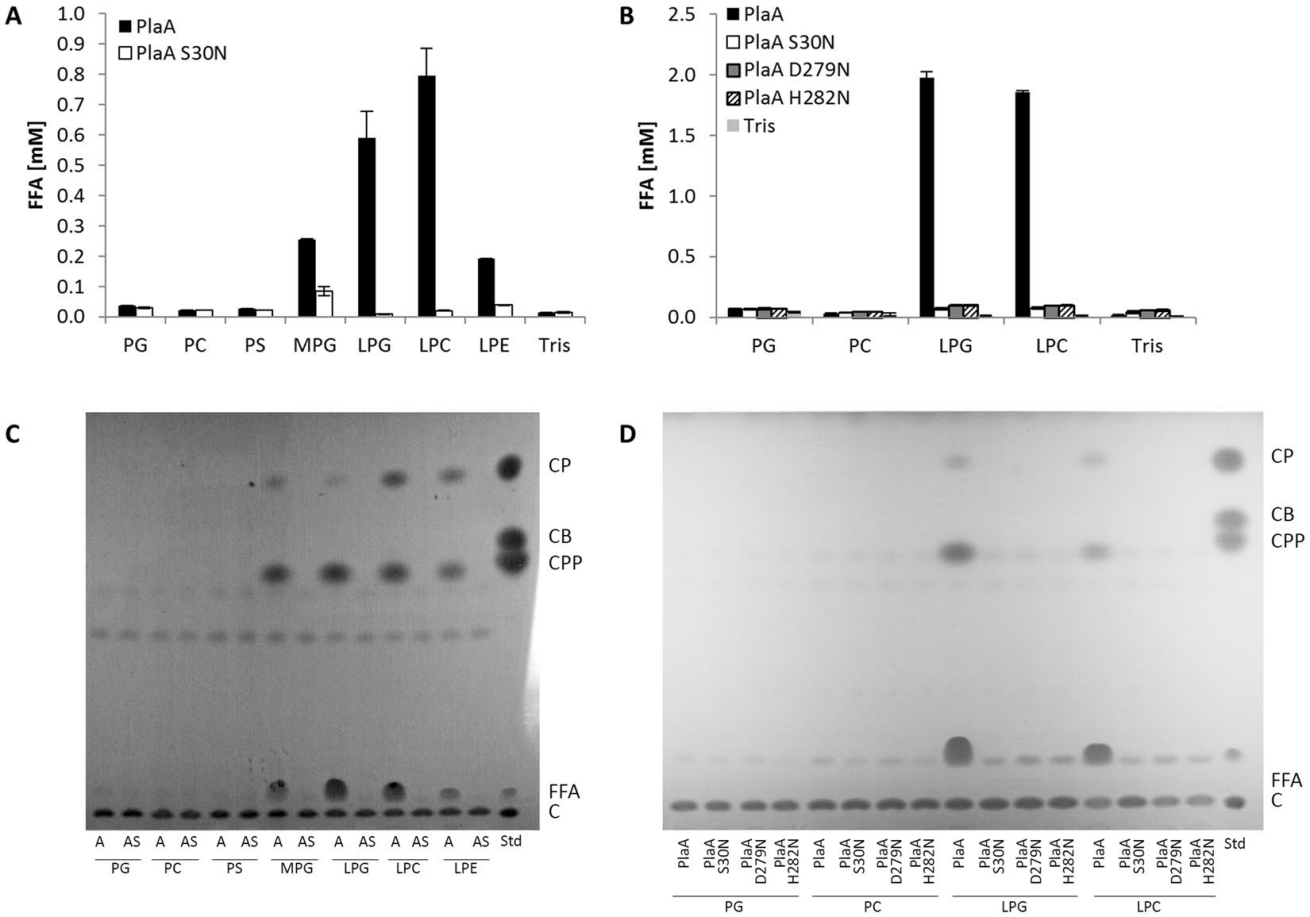
rPlaA shows LPLA, lipase, and GCAT activities which depend on the individual members of the catalytic triade serine 30, aspartate 279 and histidine 282. PLA and LPLA activites of rPlaA and catalytic site mutants were determined via quantification of FFA after incubation for 3 h with the indicated lipids at 37 °C (**A**,**B**). Formation of different cholesterol esters was studied after 16 h incubation at 37 °C with the indicated lipids, followed by lipid extraction and TLC (**C**,**D**). Reactions were performed with recombinant purified protein (**A**,**C**) or with cell lysates (**B**,**D**) of *E*. *coli* clones expressing PlaA, PlaA S30N, PlaA D279N, or PlaA H282N [BL21(pGP172 *plaA* = pCL119), BL21(pGP172 *plaA S30N* = pCL121), BL21(pGP172 *plaA D279N* = pCL122) and BL21(pGP172 *plaA H282N* = pCL123)]. The results represent the means and standard deviations of duplicate reactions (**A**,**B**). All results shown are representative for at least two additional experiments. *CP*, cholesterol palmitate; *CB*, cholesterol butyrate; *CPP*, cholesterol propionate; *FFA*, free fatty acids; *C*, cholesterol; *A*, PlaA; *AS*, PlaA S30N; *Std*, standard.

For further analysis of PlaA, the protein was recombinantly expressed in *E*. *coli* and either the purified protein or cell lysates were used for lipolysis assays. As shown in Fig. 4A, rPlaA possessed lipase and LPLA activities, the latter especially towards LPG and LPC. A site directed mutation, affecting serine 30 in the GDSL motif, as well as the other two potential members of the catalytic triade, aspartate 279 and histidine 282, reduced LPLA or lipase activities to background levels highlighting their importance in catalysis (Fig. 4A,B). GCAT activity of rPlaA was found for the transfer of short chain fatty acids (generation of CPP) and palmitic acid (generation of CP) with MPG and lysophospholipids as donors (Fig. 4C,D). The formation of CP was surprising because PlaA-dependent CP development was not found for *L*. *pneumophila* culture supernatants (Fig. 2D,E). As expected the catalytic site mutants did not exhibit GCAT activity (Fig. 4C,D). In conclusion, rPlaA shows LPLA, lipase, and GCAT activities dependent on a serine 30, aspartate 279, histidine 282 catalytic triade.

### Zinc metalloproteinase ProA boosts LPLA activity of rPlaA but diminishes GCAT activity transferring palmitic acid. PlaA is C-terminally processed by ProA

**Figure 5:**
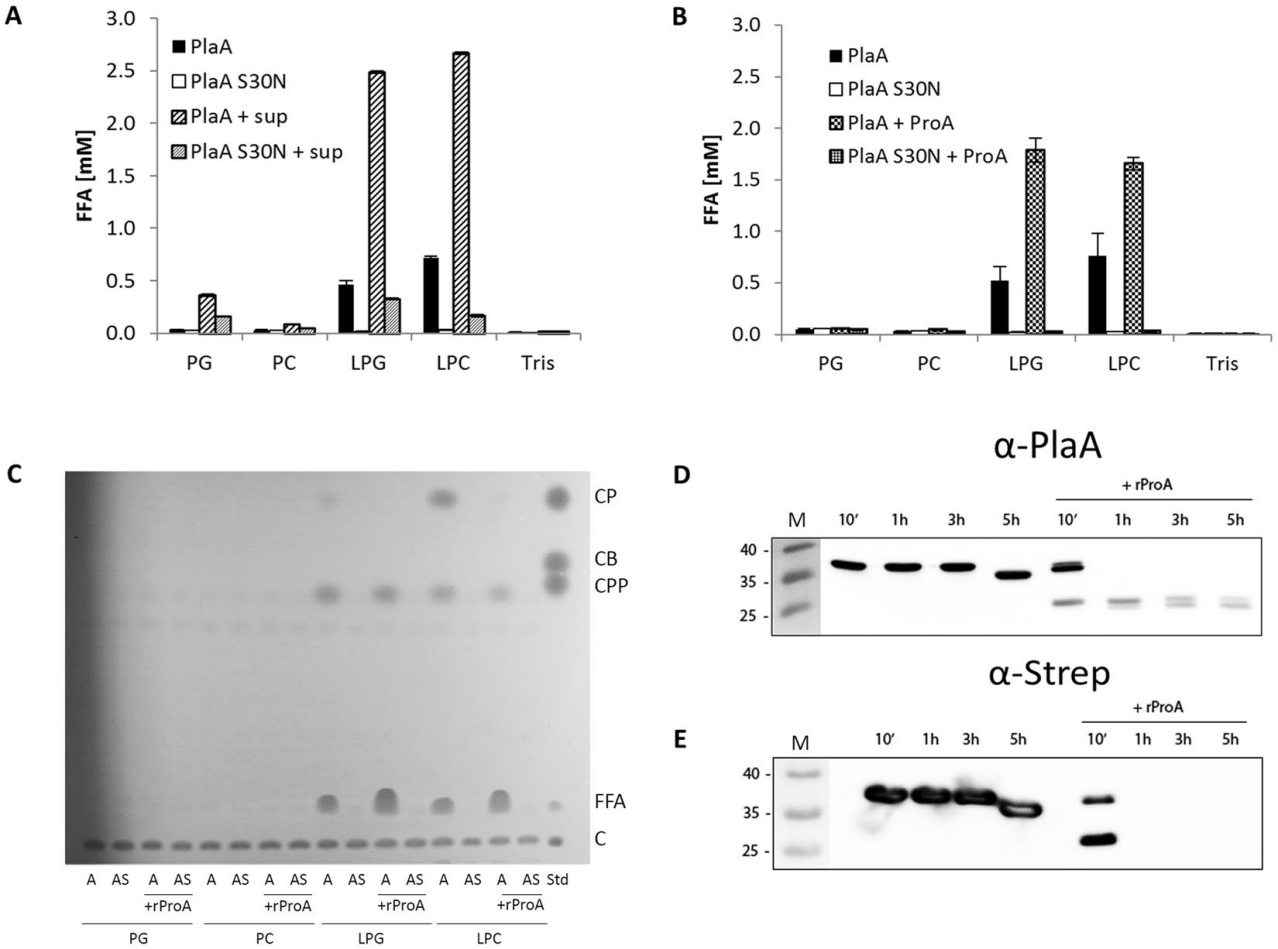
Addition of ProA increases LPLA and diminishes GCAT activity of rPlaA transferring palmitic acid. PLA and LPLA activities of purified, recombinant PlaA versus catalytic inactive PlaA S30N were determined via quantification of FFA after incubation for 3 h (**A**,**B**) with the indicated lipids at 37 °C without or with addition of either *L*. *pneumophila plaACD* ^−^ culture supernatant (**A**) or purified, recombinant ProA (**B**). Formation of different cholesterol esters by recombinant PlaA and PlaA S30N without or with addition of ProA was studied after 16 h incubation at 37 °C with the indicated lipids, followed by lipid extraction and TLC (**C**). Processing of PlaA by ProA was analyzed by anti-PlaA (**D**) anti-Strep (**E**) Western blot after incubation of rPlaA without or with rProA for indicated time points at 37 °C. Molecular weight standards (M) are shown on the left side of each panel in kDa. Blot images were cropped from all sides. Full length blots are presented in Supplementary Fig. S2. The results represent the means and standard deviations of duplicate reactions (**A**,**B**). All results shown are representative for at least two additional experiments. *CP*, cholesterol palmitate; *CB*, cholesterol butyrate; *CPP*, cholesterol propionate; *FFA*, free fatty acids; *C*, cholesterol; *A*, PlaA; *AS*, PlaA S30N; *sup*, *L*. *pneumophila plaACD* ^−^ culture supernatant.

Next we were interested if the zinc metalloproteinase ProA of *L*. *pneumophila* has an impact on PlaA activity as it has been previously demonstrated for PlaC, where especially GCAT activity increases after ProA treatment^30^. To test for a ProA-dependent effect on PlaA activity we initially used culture supernatant of an *L*. *pneumophila plaACD* mutant containing ProA but lacking GDSL enzyme-derived activities in the lipid hydrolysis samples. Indeed, a steep increase in LPLA activity with LPG and LPC as substrates was detected when the supernatant was added to the reaction mixture with purified rPlaA. In contrast, no substantial activity was observed with the purified S30N-mutated rPlaA (Fig. 5A). Of note, a slight increase in PG hydrolysis was measured in the case of incubation of rPlaA and rPlaAS30N with the culture supernatant suggesting the presence of a non-GDSL PLA in the supernatant of the *plaACD* mutant (Fig. 5A).

Western Blot analysis indicated processing of PlaA by ProA (Fig. 3) and therefore, we analyzed whether rProA also acts on rPlaA. Indeed, rPlaA but not rPlaAS30N showed a substantial increase in LPLA activity when incubated with rProA (Fig. 5B). In contrast to the earlier described increase in PlaC activity upon ProA activation, no enhancement of GCAT activity was observed for rPlaA treated with rProA. Instead, rProA addition resulted in loss of palmitic acid transfer from LPC to cholesterol (i.e. no generation of CP) by rPlaA (Fig. 5C). This might explain why no explicit PlaA-derived GCAT activity transferring palmitic acid from lysophospholipids was observed in the *L*. *pneumophila* wild type in the experiments presented above (Fig. 2D,E). In contrast, transfer of short chain fatty acids was found independent of ProA (Fig. 5C).

To verify processing of PlaA by ProA, we incubated rPlaA with rProA followed by Western blotting with a PlaA-specific antibody or another antibody directed towards the N-terminal Strep tag. In both cases, rPlaA was found to be processed after 10 minutes incubation, characterized by the appearance of an ~26–28 kDa protein band (Fig. 5D,E), which corresponds to the size of PlaA in the above analyzed *L*. *pneumophila* strains, except the *proA* mutant (Fig. 3). Cleaved rPlaA could still be detected with the anti-Strep antibody suggesting that PlaA is processed on its C-terminal side (Fig. 5D,E).

An incubation time extending 1 h resulted in stable detection of the ~26–28 kDa fragment with the PlaA-specific antibody. On the contrary, the Strep-PlaA version was no longer detectable under these conditions (Fig. 5D,E). These data suggest further processing of rPlaA at the N-terminus. However, no second substantial molecular shift of rPlaA could be observed with the PlaA antibody indicating that N-terminal cleavage of rPlaA is likely a minor procession (Fig. 5D,E). After 5 h of incubation of rPlaA without rProA further degradation of the protein from the C-terminal side occurred (Fig. 5D,E). As a conclusion, we showed that PlaA LPLA activity is steeply increased but GCAT activity is diminished by ProA which processes PlaA at its C-terminus.

### Deletion of 20 amino acids in a predicted disulphide loop of rPlaA results in boosted LPLA activity but diminished GCAT activity transferring palmitic acid

*L*. *pneumophila* PlaC GCAT is activated through truncation of a disulphide loop by ProA^30^. Like PlaC, PlaA contains a predicted disulphide loop, which in this case consists of 28 amino acids (Fig. 1). Indeed, the ~26–28 kDa PlaA fragment observed in wild type *L*. *pneumophila* culture supernatant and after treatment of rPlaA with rProA nicely matches a protein, which is cleaved within the disulphide loop region (Figs 1, 3 and 5D,E). After reducing SDS-PAGE, the disulphide bond would be opened and the cleaved C-terminal part would be released corresponding to a ~ 28 kDa fragment visible after Western blotting. A truncated disulphide loop in PlaC has been shown to render the protein more active, especially with respect to GCAT activity independently of ProA^30^.

**Figure 6:**
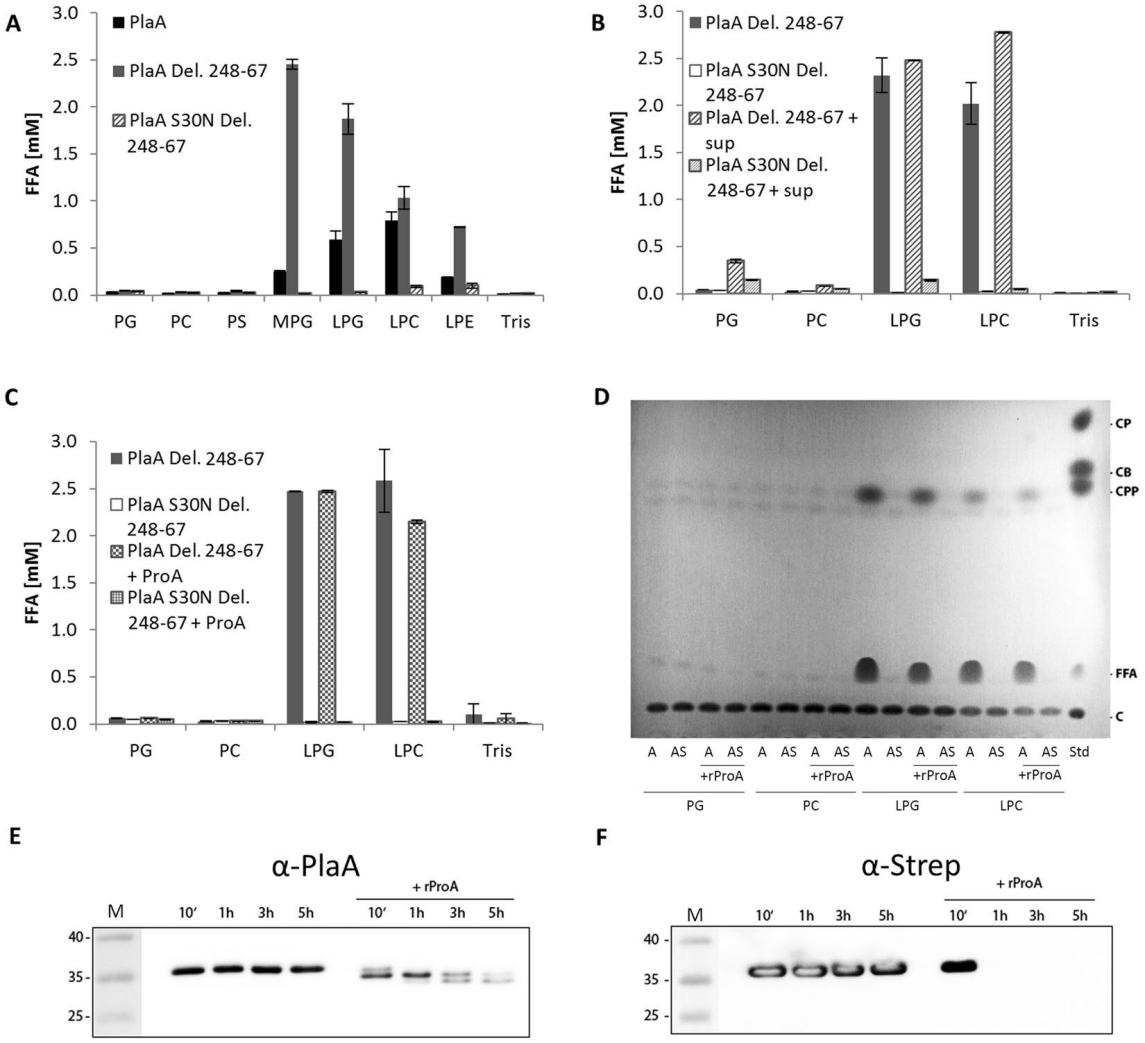
Deletion of 20 amino acids in a predicted disulphide loop of rPlaA results in increased LPLA activity and diminished GCAT activity transferring palmitic acid. PLA and LPLA activities of purified, recombinant PlaA Del. 248-67 versus catalytic inactive PlaA S30N Del. 248-67 were determined via quantification of FFA after incubation for 3 h (**A**,**B**,**C**) with the indicated lipids at 37 °C without (**A**) or with addition of either *L*. *pneumophila plaACD* ^−^ culture supernatant (**B**) or purified, recombinant ProA (**C**). Formation of different cholesterol esters by recombinant PlaA Del. 248-67 and PlaA S30N Del. 248 -67 without or with addition of ProA was studied after 16 h incubation at 37 °C with the indicated lipids, followed by lipid extraction and TLC (**D**). Processing of PlaA Del. 248-67 by ProA was analyzed by anti-PlaA (**E**) and anti-Strep (**F**) Western blot after incubation of rPlaA Del. 248-67 without or with rProA for indicated time points at 37 °C. Molecular weight standards (M) are shown on the left side of each panel in kDa. Blot images were cropped from all sides. Full length blots are presented in Supplementary Fig. S3. Results shown in Fig. 6D were from the same experiment as shown in Fig. 5C but were run on separate TLC plates. The results represent the means and standard deviations of duplicate reactions (**A**–**C**). All results shown are representative for at least two additional experiments. *CP*, cholesterol palmitate; *CB*, cholesterol butyrate; *CPP*, cholesterol propionate; *FFA*, free fatty acids; *C*, cholesterol; *A*, PlaA Del. 248-67; *AS*, PlaA S30N Del. 248-67; *sup*, *plaACD* ^−^ *L*. *pneumophila* culture supernatant.

Therefore, we deleted 20 amino acids from the disulphide loop of PlaA and analyzed lipolytic activities of purified rPlaA versus rPlaAdel248–267. Indeed, lipase and LPLA activities of the loop deletion protein increased steeply compared to full length rPlaA (Fig. 6A). When culture supernatant of the *L*. *pneumophila plaACD* mutant or rProA was added to the preparations, the degree of activation was not further enhanced indicating that in the absence of the disulphide loop region, PlaA LPLA activity is independent from ProA-based activation (Fig. 6B,C). In accordance with the results above and in contrast to steep activation of LPLA activity, GCAT activity was not detectable for the loop truncation protein, showing that GCAT activity of PlaA is diminished by loop deletion (Fig. 6D). For the loop deletion protein no substantial processing after ProA addition, as it was observed for rPlaA (Fig. 5D,E), was detected with the PlaA or Strep tag antibodies. However, a limited N-terminal processing releasing the N-terminal Strep-Tag was observed (Fig. 6E,F). This means that deletion of the disulphide loop leads to comparable activities as seen for full length PlaA processed by ProA. We therefore localized the ProA cleavage site within PlaA to the amino acid region between aspartate 248 and leucine 267.

### PlaA is cleaved by ProA between glutamate 266 and leucine 267

**Figure 7:**
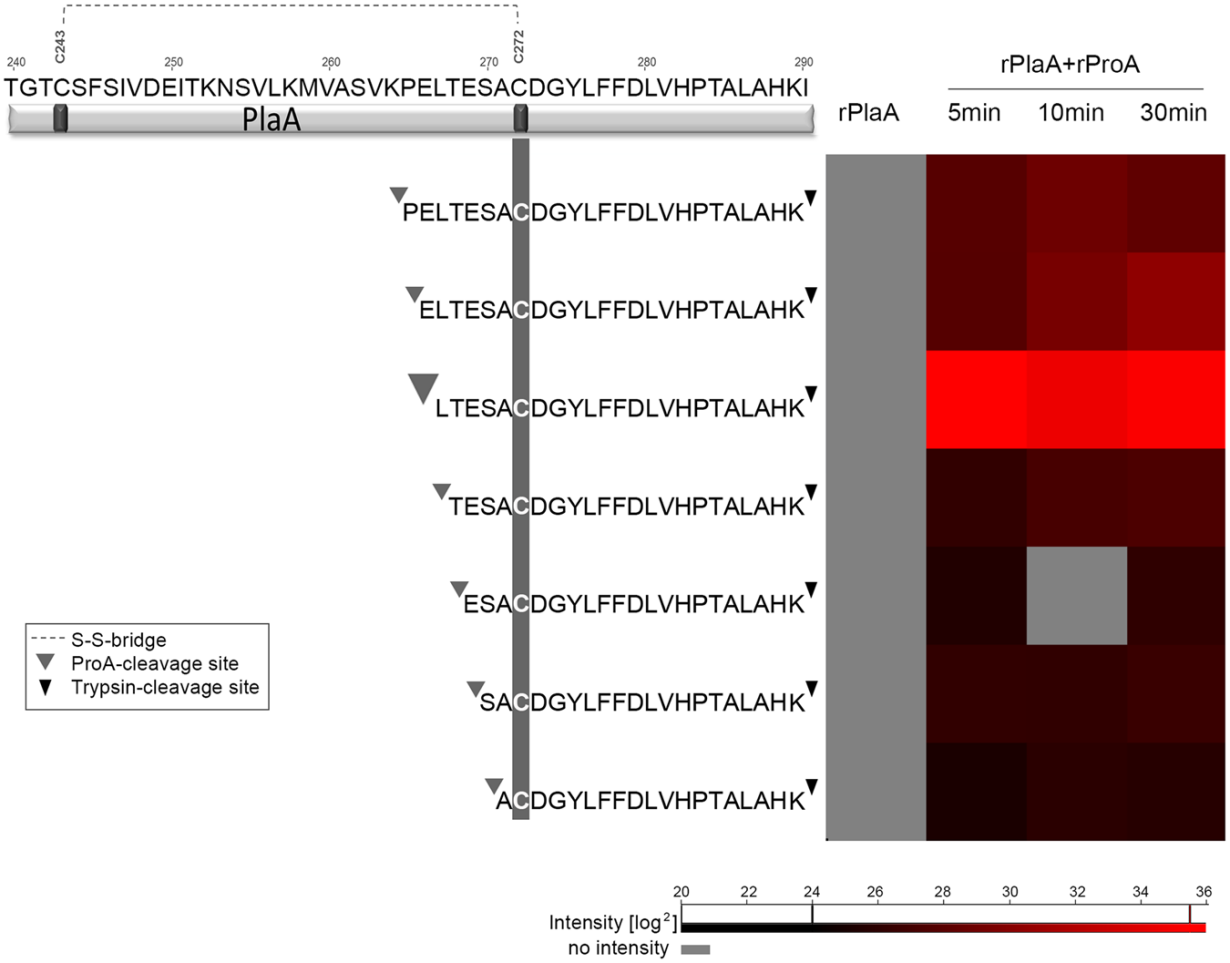
Overview of semi-tryptic peptides of PlaA cleaved by ProA identified by mass spectrometry. The upper part displays a fragment of the PlaA protein sequence (amino acid 240 to 290). The heat map on the right site visualizes the intensity values of the peptides found cleaved by ProA, whereby brighter red represents a higher intensity. All potential cleavage sites were detected within the disulfide loop region between cysteine 243 and cysteine 272. The most probable ProA-cleavage site within PlaA is in front of leucine 267 indicated by the big grey arrow. Less probable cleavage sites are highlighted by a smaller grey arrow. Small black arrows indicate trypsin cleavage sites.

Mass spectrometry analysis of rPlaA incubated with rProA indeed confirmed that PlaA is cleaved by ProA between cysteine 243 and cysteine 272. The peptides identified indicated that all potential cleavage sites are within the disulphide loop region. The most probable ProA-cleavage site within PlaA was in front of leucine 267 because the respective peptide displayed the highest intensity (Fig. 7, Table. S 1). Mutagenesis of glutamate 266 and leucine 267 to asparagine was not sufficient to prevent the cleavage of PlaA by ProA (Fig. S 4). This indicates that also less preferential sites may be cleaved by ProA which then also results in protein activation.

## Discussion

We previously described that a GDSL enzyme of *L*. *pneumophila*, PlaC, is activated by a ProA-dependent disulphide loop cleavage. After cleavage, the two fragments of the protein, each containing catalytic essential amino acids, are kept together by a disulphide bond (Fig. 1). PlaC cleavage by ProA predominantly activates GCAT and also PLA activity of the enzyme. Mutants of PlaC where the disulphide loop was shortened from 44 to 31, 24, 22, or 6 amino acids show ProA-independent activation of GCAT, suggesting a loop-mediated enzyme inhibition/activation mechanism. This ProA-mediated regulation might serve to protect the bacterium from a potential harmful activity of PlaC within the cell^30^.

A variety of enzymes with distinct activities using similar but not identical activation strategies have been described before. For example, *Pseudomonas* exotoxin A is a ADP-ribosylase, which is activated by proteolytic cleavage within a disulphide loop and subsequent reduction of the disulphide bridge, thereby releasing the active domain^39,40^. Another example is Shiga toxin of enterohemorrhagic *E*. *coli*, where the C-terminal region of subunit A contains a disulphide loop with the trypsin and furin recognition sequence R-V-A-R. Cleavage results in enzymatically active N-glycosidase and disulphide bond linked A1 and A2 fragments^41,42^.

**Figure 8:**
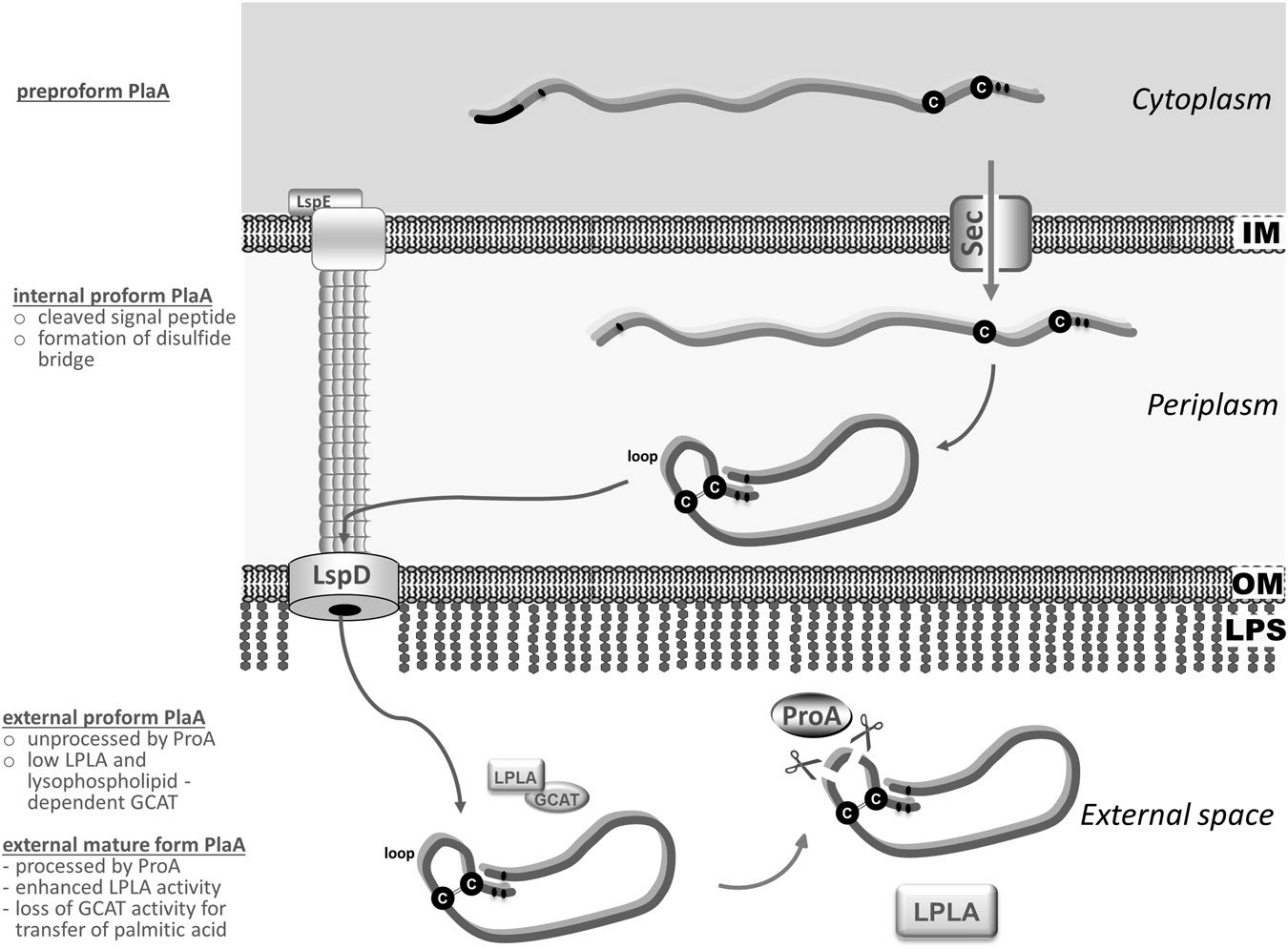
Proposed model for export and ProA-dependent processing of PlaA. PlaA and ProA are present in a preproform within the cytoplasm. Both are transported through the inner bacterial membrane via the Sec system and concomitant signal peptide cleavage. In the periplasm, a disulphide bond may be formed within PlaA (internal preform). Quantities and PlaA-derived activity in association with the bacterial cell are very low (Figs 2C and 3) compared to those in the external space suggesting fast export of the protein. After subsequent export of PlaA and ProA via the type II secretion system Lsp, ProA acquires its mature form via autoactivation by means of N-terminal propeptide cleavage^43^. PlaA (external preform) is then processed by ProA resulting in the external mature form of PlaA with increased LPLA activity and lost GCAT activity for transfer of palmitic acid. *IM*, inner bacterial membrane; *OM*, outer bacterial membrane; *LPS*, lipopolysaccharide.

In the case of PlaA, a ProA-dependent activation via disulphide loop processing was found and we propose the following model of PlaA activation. PlaA and ProA are both present as preproenzymes within the cytoplasm. Both are transported through the inner bacterial membrane via the Sec system and concomitant signal peptide cleavage. In the periplasm, a disulphide bond may be formed within PlaA (internal preform). Cell-associated quantity and activity of PlaA are very low (Figs 3 and 2C) compared to those in the external space suggesting fast export of the protein. After subsequent export of PlaA and ProA via the type II secretion system Lsp, ProA acquires its mature form via autoactivation by means of N-terminal propeptide cleavage^43^. PlaA (external preform) is then processed by ProA resulting in the external mature form of PlaA with increased LPLA activity and lost GCAT activity for transfer of palmitic acid (Fig. 8).

LPLA activity steeply increased when PlaA was processed by ProA or alternatively when the disulphide loop was shortened from 28 to 8 amino acids. We previously described that the loop region of PlaC shared substantial homology to PlaC-like enzymes from *Legionella* but not to the corresponding PlaA-like enzymes^30^. Similarly, the loop region in PlaA is only conserved in PlaA-like enzymes of *L*. *pneumophila*. Mass spectrometry analysis revealed that PlaA is cleaved by ProA within the loop region, but mutagenesis of the most probable cleavage site was not sufficient to prevent the digestion by ProA. The same was observed for PlaC, where several ProA-cleavage sites were found within the disulphide loop region, but mutagenesis studies of potential target sites indicated that the protease either is not very selective or that structural features are important^30^.

Although the proteolytic processing is very similar between PlaC and PlaA they are differentially regulated by ProA. Unprocessed PlaA possesses LPLA activity and has specific GCAT activity, only accepting lysophospholipids as fatty acid donors. Unprocessed PlaC has PLA activity and corresponding GCAT activity, which transfers palmitic acid and short chain fatty acids from diacylphospholipids to cholesterol. While PLA and GCAT activity of PlaC are both enhanced by proteolytic processing, PlaA processing leads to enhanced LPLA activity but diminished GCAT activity. We conclude that external proPlaA may predominantly act as GCAT at earlier time points of growth prior to ProA secretion. However, at later time points after ProA-mediated processing, PlaA shifts its activity to a very potent LPLA (Figure 8). Activated PlaA may then contribute to phagosomal membrane disintegration as described before when the protective type IVB secreted Dot/Icm effector SdhA is not present^37^.

Most interestingly, the type IVB secreted effector SdhA counteracts LCV disintegration by type II-secreted PlaA^37^. The mechanism by which this is achieved and whether the interaction is direct or indirect is not known so far. Nevertheless, keeping the vacuole membrane intact is a very important process to prevent immune detection of *L*. *pneumophila* ^37,44^. It is possible that PlaA acts from the inside of the phagosome whereas the Dot/Icm injected SdhA rather acts from the outside. One can hypothesize that PlaA, although not able to drive bacterial exit from the LCV in the presence of SdhA, prepares the membrane for facilitated exit at a later time point under the influence of additional factors. Further, PlaA might help to modify the LCV membrane in a way that it is more prone to fuse with other lipid compartments or may facilitate expansion of the phagosomal membrane when the bacteria are replicating.

But the question remains: How does an LPLA activity, directed towards lysophospholipids but not diacylphospholipids, disintegrate the LCV membrane? The type II-secreted enzyme ProA was previously found in the LCV lumen^36^; therefore it is possible that other type II secreted enzymes, such as PlaA or PlaC, localize to the same compartment. Provided that the LCV membrane resembles the membrane of the endoplasmic reticulum or the plasma membrane, its major lipid components would be PC, phosphatidylethanolamine, phosphatidylserine, and phosphatidylinositol^45^. In this case an LPLA alone should not substantially disintegrate the membrane of the compartment. Nevertheless, several conditions may facilitate such a process: specifically when 1) in addition to ProA another yet unknown activating factor switches LPLA to PLA activity, 2) the LCV contains a substantial amount of lysophospholipids or another yet not identified substrate of PlaA, and 3) PlaA acts together with another enzyme showing PLA activity. The latter point seems most plausible. PlaC or another currently not identified PLA could potentially catalyze the first step resulting in the release of a fatty acid from a diacylphospholipid thereby producing the lysophospholipid substrate for PlaA. PlaC was found in *L*. *pneumophila* OMVs but not PlaA^34^. If a twostep hydrolysis of the LCV membrane is conceivable by PlaC and PlaA, fusion of PlaC-containing OMVs might strongly promote the disintegration process^34^. PlaA may then take over the second step and hydrolyze the lysophospholipid and thereby may accelerate the process substantially. Furthermore, enhanced LPLA activity could be useful for the elimination of the signal transducer LPC^46^. LPC is known as a factor for enhancement of neutrophil bactericidal activity and can lead to reduced bacterial viability^47,48^. Lower LPC levels as a result of increased LPLA activity might therefore support bacterial survival and integrity.

## Methods

### Bacterial strains and growth conditions

*L*. *pneumophila* sg1 strain Corby was used for all experiments, including knock out mutant construction, as well as for recombinant gene expression in *E*. *coli* strains DH5α and BL21. The used strains are listed in Table 1 and were grown on buffered charcoal yeast extract (BCYE) agar and in buffered yeast extract (BYE) broth (both for *Legionella*), or on Luria Bertani (LB) agar and in LB broth (both for *E*. *coli*) as described previously^28,49,50^.

**Table 1:** Overview of strains used in this study.

Organism	Mutation(s)/Plasmid	Tags	selection marker(s)	Reference
*L*.*pn*. Corby	wild type		-----	62
*L*.*pn*. Corby	*plaA* knock out		Km^R^	30
*L*.*pn*. Corby	*plaC* knock out		Km^R^	30
*L*.*pn*. Corby	*plaD* knock out		Km^R^	30
*L*.*pn*. Corby	*plaACD* knock out		Km^R^, Hyg^R^, Gent^R^	this study
L.pn. Corby	proA knock out		Km^R^	30
L.pn. Corby	lspDE knock out		Km^R^	32
L.pn. Corby	lssB knock out		Km^R^	63
L.pn. Corby	fliA knock out		Km^R^	64
L.pn. JR32	wild type		-----	65
L.pn. JR32	dotA knock out		Km^R^	66
L.pn. JR32	dotB knock out		Km^R^	65
L.pn. JR32	lvh knock out		Gent^R^	67
*E. coli* BL21	pCL119 = pGP172 plaA_Corby_ (-SP*)	N-term. Strep-tag	Amp^R^	this study
*E. coli* BL21	pCL121 = pGP172 plaA_Corby_ S30N (-SP)	N-term. Strep-tag	Amp^R^	this study
*E. coli* BL21	pCL122 = pGP172 plaA_Corby_ D279N (-SP)	N-term. Strep-tag	Amp^R^	this study
*E. coli* BL21	pCL123 = pGP172 plaA_Corby_ H282N (-SP)	N-term. Strep-tag	Amp^R^	this study
*E. coli* BL21	pCL126 = pGP172 plaA_Corby_ ΔAA248-67(-SP)	N-term. Strep-tag	Amp^R^	this study
*E. coli* BL21	pCL127 = pGP172 plaA_Corby_ S30N ΔAA248-67 (-SP)	N-term. Strep-tag	Amp^R^	this study
*E. coli* BL21	pMH41 = pGP172 plaA_Corby_ E266N L267N	N-term. Strep-tag	Amp^R^	this study
*E. coli* BL21	pSB2 = pGP172 plaD_Corby_	N-term. Strep-tag	Amp^R^	this study
*E*. *coli* BL21	pCL15 = pet28a(+) *proA*_Corby_	C-term. 6xHis-tag	Km^R^	30
*E*. *coli* DH5α	pCL7 = pBCKS P_*plaD*_ *plaD*_Corby_		Cm^R^ this study	
*E*. *coli* DH5α	pCL13 = pBCKS P_*plaD*_ *plaD*_Corby_::Hyg^R^		Hyg^R^, Cm^R^	this study
*E*. *coli* DH5α	pBH1 = pGEMTez *plaC*_130b_		Amp^R^	28
*E*. *coli* DH5α	pER11 = pGEMTez *plaC*_130b_::Gent^R^		Gent^R^, Amp^R^	this study

*[-SP]: constructs cloned without sequence coding for the putative signal peptide (aa 1–18)].

### DNA techniques and sequence analysis

*E*. *coli* BL21 was used for the propagation of recombinant plasmid DNA with backbones of the following vectors: pGP172^51^ and pet28a(+) (Novagen). Genomic and plasmid DNA were prepared, amplified, and sequenced according to standard protocols. Primers were obtained from Eurofins MWG Operon. Restriction enzymes were purchased from New England Biolabs. Foreign DNA was introduced into *E*. *coli* strains by electroporation with a Life Technologies cell porator according to the manufacturer’s specifications as described earlier^28^. Nucleotide and translated protein sequences were analyzed using the Geneious 10.0.05^52^, the NCBI website (http://www.ncbi.nlm.nih.gov/), DiANNA 1.1 (http://clavius.bc.edu/~clotelab/DiANNA/), SignalP 4.1 (http://www.cbs.dtu.dk/services/SignalP/) and ExPASy (http://www.expasy.ch).

### Cloning of *L. pneumophilaplaA* and *plaD* into pGP172 for recombinant expression in *E. coli*

*L*. *pneumophila* Corby *plaA (lpc1811) and plaD (lpc 0558)* were cloned into the pGP172 vector resulting in pCL119 and pSB2 (N-term. Strep-Tag). To create PlaA disulphide loop deletion mutant constructs, pCL119 was amplified with primers listed in Table 2 and ligated; resulting in pCL126. To introduce mutations into the *plaA* gene, the pCL119 vector was mutated by means of the QuikChange Site-Directed Mutagenesis Kit (Stratagene) with the primers listed in Table 2, resulting in pCL121-pCL123, pCL127 and pMH41.

**Table 2:** Overview of primers used in this study.

Plasmid	Gene	Tag	Primer name	Primer sequence
pCL119	*plaA*_*Corby*_ (-SP)	N-term Strep-Tag	plaA-SP_SacI_fw	5′-TAGAGCTCTATGACACCACTTAATAACATAG-3′
			plaA-BamHI_rv	5′-TAGGATCCTTAATTCTCGGCGAA-3′
pCL121	*plaA*_*Corby*_ S30N (-SP)	N-term Strep-Tag	PlaA_S30N_fw	5′-GTATTTGGTGATAATTTGTCGGATAACGG-3′
			PlaA_S30N_rv	5′-CCGTTATCCGACAAATTATCACCAAATAC-3′
pCL122	plaA_Corby_ D279N (-SP)	N-term Strep-Tag	PlaA_D279N_fw	5′-GGTTATTTGTTTTTTAATTTGGTTCATCCGACA-3′
			PlaA_D279N_rv	5′-TGTCGGATGAACCAAATTAAAAAACAAATAACC-3′
pCL123	plaA_Corby_ H282N (-SP)	N-term Strep-Tag	PlaA_H282N_fw	5′-TTTGATTTGGTTAATCCGACAGCGTTG-3′
			PlaA_H282N_rv	5′-CAACGCTGTCGGATTAACCAAATCAAA-3′
pCL126	plaA_Corby_ ΔAA248-67 (-SP)	N-term Strep-Tag	PlaA_Del267_fw	5′-ACAGAAAGCGCATGTGATGGT-3′
			PlaA_Del248_rv	5′-TATCGAAAAGGAGCAAGTACCTGT-3′
pCL127	plaA_Corby_ S30N ΔAA248-67 (-SP)	N-term Strep-Tag	PlaA_S30N_fw	5′-GTATTTGGTGATAATTTGTCGGATAACGG-3′
			PlaA_S30N_rv	5′-CCGTTATCCGACAAATTATCACCAAATAC-3′
pCL7	P_*plaD*_ *plaD*_Corby_		PromD270_SacI	5′-GAGAGCTCTCCGTGCTCTTGCTGATA-3′
			PlaD_r_XhoI	5′-GACTCGAGATTAAGTTAGGATTTCGTT-3′
pCL13	Hyg^R^		Hyg-HpaI-fw	5′-AATTTAGTTAACATGACACAAGAATCCCTG-3′
			Hyg-HpaI-rv	5′-AATTTAGTTAACTCAGGCGCCGGGGGC-3′
pER11	Gent^R^		BamH_Gent1	5′-AAGGATCCGACGCACACCGTGGAAA-3′
			HindGent2	5′-TCGGAAGCTTGCGGCGTTGTGACAATTT-3′
pSB2	*plaD*_*Corby*_	N-term Strep-Tag	PlaD_SacI_fw	5′-ATGAGCTCAATGGCCCAAAAA-3′
			PlaD_BamHI_rv	5′-ATGGATCCTCAGGTAAATTTAAC-3′
pMH41	plaA_Corby_ E266N L267N		PlaA_E266N_L267N_fw	5′-TCTGTGAAGCCAAATAATACAGAAAGCGCA-3′
			PlaA_E266N_L267N_rv	5′-TGCGCTTTCTGTATTATTTGGCTTCACAGA-3′

### *L. pneumophila* GDSL enzyme knock out mutant construction

Plasmids pER11 and pCL13, containing the *plaC and plaD* genes, disrupted or partially replaced by a Gent^r^ and Hyg^r^ cassette, respectively, were used to introduce Gent^r^ and Hyg^r^ gene insertion mutation into the chromosome of strain *L*. *pneumophila* Corby *plaA*-mutant^30^ via allelic exchange. To generate pER11, *L*. *pneumophila* 130b *plaC* was cloned into the pGEM-Tez vector yielding in pBH1^28^. After pBH1 restriction by means of HindIII and BamHI, the Gent^r^-gene cassette was cloned into the pBH1 vector yielding in pER11 (primers see Table 2). To generate pCL13, *L*. *pneumophila* Corby *plaD* was cloned into the pBCKS vector yielding in pCL7 (primers see Table 2). After the digestion of pCL7 with HpaI, the Hyg^r^-gene cassette was cloned into the vector yielding in pCL13 (primers see Table 2). Knockout mutants were generated by natural transformation and homologous recombination^29,53^. PCR was used to examine Gent^r^ and Hyg^r^ *Legionella* for the respective mutation.

### Preparations of culture supernatants and cell lysates

For assessment of hydrolytic activities, bacteria were harvested at the end of exponential growth (if not stated otherwise at an OD_600_ of about 4). Culture supernatants were obtained by centrifugation for 5 min at 5000 g and in some instances were subsequently concentrated by ultra-filtration with an exclusion size of 10 kDa. Cell lysates were produced as described previously^29,54^. Culture supernatants and cell lysates were tested immediately for enzymatic activities.

### Purification of recombinant ProA

Recombinant periplasmic ProA was isolated by osmotic shock^55^ from *E*. *coli* BL21(pCL15) and subsequent anion exchange chromatography as described before^30^.

### Overexpression of recombinant Strep-PlaA, Strep-PlaA variants, and Strep-PlaD

For expression of recombinant Strep-PlaA (from pCL119), Strep-PlaA variants (from pCL126-127) and Strep-PlaD (from pSB2), *E*. *coli* BL21 cultures were grown at 37 °C to an OD_600_ = 0.8, induced with 0.1 mM IPTG and transferred to 18 °C for 16-17 h. For the purification of Strep-PlaA, Strep-PlaA variants and Strep-PlaD, the cell pellet was collected and resuspended in 100 mM Tris-HCl pH 8.0, containing 100 mM NaCl and 1 mM EDTA. Homogenization was performed by Emulsi Flex C3 (Avestin) applying 25,000 to 30,000 psi. The soluble fraction was isolated by centrifugation at 13,000 g and 4 °C for 60 min. Strep-PlaA and variants were purified using a Strep-Tactin^®^ Superflow^®^ high capacity resin (IBA) according to the manufacturer’s instructions.

### Assays for lipolytic activities

Lipolytic (PLA, LPLA, lipase, acyltransferase/GCAT) activities were detected as described previously^28,29,56^ by using the lipid substrates 1-monopalmitoyllyso-phosphatidylcholine (LPC), 1—mono-palmitoyllysophosphatidylglycerol (LPG), 1-monopalmitoylglycerol (MPG), monopalmitoyllysophosphatidylethanolamine (LPE), dipalmitoylphosphatidylserine (PS), dipalmitoylphosphatidylethanol-amine (PE), 1,2—dipalmitoylphosphatidyl-glycerol (PG), 1,2-dipalmitoylphosphatidylcholine (PC) and cholesterol. In brief, 25 ng Strep-PlaA, Strep-PlaA-variants or 25 µl *E*. *coli* cell lysates were incubated with the lipid suspension and with or without 3.5 mUnit ProA or 25 µl supernatant from *L*. *pneumophila plaACD* ^*-*^ in a total reaction volume of 50 µl. For GCAT activity the reaction mix was doubled and cholesterol was added. All lipids, including standards for thin layer chromatography (TLC) were obtained from Sigma Chemicals or Avanti Polar Lipids. Detection of reaction products, such as free fatty acids and sterol esters, was performed by lipid extraction and subsequent TLC or by quantitative detection of free fatty acids as described earlier^28,29^.

### Processing of recombinant PlaA by recombinant ProA

250 ng Strep-PlaA or Strep-PlaA E266N L267N was incubated with and without 0.5 mU recombinant ProA for 10 min, 1 h, 3 h, and 5 h. Afterwards, the samples were examined by Western Blot analysis using primary polyclonal rabbit antibody against PlaA and monoclonal HRP-conjugated mouse StrepMAB-Classic antibody (IBA) against Strep-Tag.

### Western Blot analysis

Samples were separated on a 12.5% SDS-polyacrylamide gel^57^. Proteins were transferred to a nitrocellulose membrane. Primary polyclonal rabbit antibodies against PlaA, PlaD (generated by BioGenes against recombinant expressed and purified PlaA and PlaD) and PlaC^30^ were added at a dilution of 1:1000. α-rabbit linked to G-horseradish peroxidase (Sigma) was used as secondary antibody diluted 1:10,000. For detection of the Strep-Tag the monoclonal mouse StrepMAB-Classic antibody (IBA) was used at a dilution of 1:5000. Proteins were detected by chemiluminescence (ECL Kit; Amersham).

### Determination of the ProA cleavage site within PlaA by mass spectrometry

Sample preparation: 5 µg Strep-PlaA was incubated with and without 5mU ProA for 5, 10 and 30 min. The reaction mix was stopped by the addition of 1% SDS and 5 min at 95 °C. The protein was precipitated by 4 volumes of pre-cooled acetone for 1 h at −20 °C. The dried protein pellets were resuspended in 1 M urea, 100 mM Tris⋅Cl, pH 8.5 and digested for 16 h at 37 °C using Trypsin Gold, mass spectrometry grade (Promega, Fitchburg, WI, USA) at a protein/enzyme ratio of 50:1. Peptides were reduced and alkylated in 10 mM Tris(2-carboxyethyl)phosphine and 40 mM 2-chloroacetamide at 99 °C for 5 min and further desalted using 200 µL StageTips packed with one Empore™ SPE Disk C18 (3 M Purification, Inc., Lexington, USA)^58^.

nLC-MS/MS: peptides were analyzed on an EASY-nanoLC 1200 (Thermo Fisher Scientific, Bremen, Germany) coupled online to a Q Exactive™ Plus mass spectrometer (Thermo Fisher Scientific, Bremen, Germany). 100 ng peptides were loaded on a Acclaim™ PepMap™ trap column (20 mm × 75 μm i.d., 100 Å, C18, 3 μm; Thermo Fisher Scientific, Bremen, Germany) at a flow rate of 3 µL/min for 5 min and were subsequently separated on a 50 cm Acclaim™ PepMap™ column (75 μm i.d., 100 Å C18, 2 μm; Thermo Fisher Scientific, Bremen, Germany) using a linear 70 min gradient of 2 to 50% acetonitrile in 0.1% formic acid at 200 nL/min flow rate. Column temperature was kept at 60 °C using a butterfly heater (Phoenix S&T, Chester, PA, USA). The Q Exactive™ Plus was operated in a data-dependent manner in the m/z range of 300–1,650 with a resolution of 70,000 using an automatic gain control (AGC) target value of 5 × 105 with a maximum injection time of 50 ms. Up to the 12 most intense 2 + - 6 + charged ions were selected for higher-energy c-trap dissociation (HCD) with a normalized collision energy of 25%. Fragment spectra were recorded at an isolation width of 1.5 Th and a resolution of 17,500@200 m/z using an AGC target value of 1 × 105 with a maximum injection time of 110 ms. The minimum MS² target value was set to 1 × 104. Once fragmented, peaks were dynamically excluded from precursor selection for 30 s within a 10 ppm window. Peptides were ionized using electrospray with a stainless steel emitter, I.D. 30 µm, (Proxeon, Odense, Denmark) at a spray voltage of 2.1 kV and a heated capillary temperature of 275 °C.

### Data analysis

The mass spectra were analyzed using MaxQuant (Version 1.5.1.2)^59^. At first, parent ion masses were recalibrated using the ‘software lock mass’ option^60^ before the MS² spectra were searched using the Andromeda algorithm against the ProA and PlaA sequences as well as 245 entries of the cRAP database (http://www.thegpm.org/crap/). Spectra were searched with a tolerance of 4.5 ppm in MS1 and 20 ppm in HCD MS² mode, using semi-tryptic specificity (KR not P) and allowing up to one missed cleavage site. Cysteine carbamidomethylation was set as a fixed modification and methionine oxidation as a variable modification. The false discovery rate was set to 1% for peptide and 1% protein identifications. Identifications were transferred between samples using the ‘match between run’ option within a match window of 0.7 min and an alignment window of 20 min. The statistical analysis of the MaxQuant results was done in Perseus (Version 1.5.0.31)^61^. At first, reverse peptide hits and contaminants were removed. Semi-tryptic peptides of PlaA with no intensities in the PlaA-only control are potential cleavage sites of ProA and were visualized in a heatmap based on their intensity values in the peptides.txt file.

### Figure preparation

Figures were prepared with Microsoft Power Point 2010. All images, symbols and fonts within the figures are acquired from Microsoft Power Point 2010. Western Blots and TLCs were labelled and processed with Adobe Photoshop CS6. Graphs included in the Figures are visualized by Microsoft Exel 2010.

### Data Availability

All data generated or analysed during this study are included in this published article or in the supplementary information (Figs S 1–4 and Table S 1).

## Electronic supplementary material

Supplementary information
