# Supplementary material for: Disulfide loop cleavage of Legionella pneumophila PlaA boosts lysophospholipase A activity
Source: Sci Rep. 2017 Nov 24;7:16313. doi: 10.1038/s41598-017-12796-4 (PMC5701174; doi:10.1038/s41598-017-12796-4)
Supplement: Supplementary file 1 — Supplementary information [file 41598_2017_12796_MOESM1_ESM.docx]

**Disulfide loop cleavage of Legionella pneumophila PlaA boosts lysophospholipase A activity**

Christina Lang^§^, Miriam Hiller^§^, Antje Flieger*

*correspondence should be addressed to: [fliegera@rki.de](mailto:fliegera@rki.de)

^§^authors contributed equally

**Supplementary information**

supernatants cell lysates





**α-PlaD**

**α-PlaA**

**α-PlaC**

**Fig. S1: Full length blots depicted in Fig. 3.** Western blot analysis and detection with α-PlaA (A-D), α-PlaC (E-H), and α-PlaD (I-L) antibodies. Results are representative for at least two additional experiments. Molecular weight standards (M) are shown in figures A, C, E, G, I and K in kDa. (X=data not relevant for this manuscript)





**Fig. S2: Full length blots depicted in Fig. 5.** Processing of rPlaA by rProA was analyzed by anti-PlaA (A, B) and anti-Strep (C, D) Western blot after incubation of rPlaA without or with rProA for indicated time points at 37 °C. Molecular weight standards (MW) are shown in figures A and B while detected signals are shown in figures C and D.

**

**

**Fig. S3:** **Full length blots depicted in Fig. 6.** Processing of rPlaA Del. 248-67 by rProA was analyzed by anti-PlaA *(A)* anti-Strep *(B)* Western blot after incubation of rPlaA Del. 248-67 without or with rProA for indicated time points at 37 °C. Molecular weight standards (MW) are shown in figures A and B while detected signals are shown in figures C and D.

**

**

**Fig. S4:** **Processing of rPlaA E266N L267N by rProA analyzed by anti-PlaA** **Western blot**. rPlaA E266N L267N was incubated without or with rProA for indicated time points at 37 °C. Molecular weight standards (MW) are shown in figure A while detected signals are shown in figure B.

**Table S1: Extract from peptides.txt output table calculated by Max Quant displaying semi-tryptic peptides of PlaA cleaved by ProA.**

| **PlaA Intensity [log2]** | **PlaA+ProA 5min Intensity [log2]** | **PlaA+ProA 10min Intensity [log2]** | **PlaA+ProA 30min Intensity [log2]** | **posterior error probability** | **Andromeda Score** | **Missed cleavages** | **Start position** | **End position** | **Sequence** | **Proteins** |
| --- | --- | --- | --- | --- | --- | --- | --- | --- | --- | --- |
| NaN | 27.8839 | 28.9034 | 28.295 | 6.15E-182 | 294.26 | 0 | 265 | 289 | PELTESACDGYLFFDLVHPTALAHK | P0\|Strep-PlaA OS=Legionella pneumophila Corby GN=PlaA |
| NaN | 27.8506 | 29.3819 | 30.4002 | 2.32E-87 | 222.48 | 0 | 266 | 289 | ELTESACDGYLFFDLVHPTALAHK | P0\|Strep-PlaA OS=Legionella pneumophila Corby GN=PlaA |
| NaN | 35.9443 | 34.634 | 35.2785 | 0 | 461.48 | 0 | 267 | 289 | LTESACDGYLFFDLVHPTALAHK | P0\|Strep-PlaA OS=Legionella pneumophila Corby GN=PlaA |
| NaN | 26.1982 | 27.2113 | 27.4281 | 3.34E-27 | 129.02 | 0 | 268 | 289 | TESACDGYLFFDLVHPTALAHK | P0\|Strep-PlaA OS=Legionella pneumophila Corby GN=PlaA |
| NaN | 25.5035 | NaN | 26.1253 | 6.00E-11 | 89.011 | 0 | 269 | 289 | ESACDGYLFFDLVHPTALAHK | P0\|Strep-PlaA OS=Legionella pneumophila Corby GN=PlaA |
| NaN | 26.2251 | 26.1456 | 26.5832 | 0.00033363 | 69.361 | 0 | 270 | 289 | SACDGYLFFDLVHPTALAHK | P0\|Strep-PlaA OS=Legionella pneumophila Corby GN=PlaA |
| NaN | 25.1812 | 25.9033 | 25.7231 | 0.0016124 | 65.808 | 0 | 271 | 289 | ACDGYLFFDLVHPTALAHK | P0\|Strep-PlaA OS=Legionella pneumophila Corby GN=PlaA |

NaN=Not a Number=no intensity
